# Effectiveness of Online Traditional Exercise Program on the Stress Level of Elderly

**DOI:** 10.1155/jare/9332171

**Published:** 2024-11-28

**Authors:** Suwat Jitdamrong, Kunlasatree Jumpathong, Choladda Nanglad, Yadaporn Ketkaeo, Dolrada Malicum, Ormjai Taejarernwiriyakul

**Affiliations:** ^1^Department of Physical Therapy, Faculty of Physical Therapy, Srinakharinwirot University, Ongkharak Campus, Nakhon Nayok 26120, Thailand; ^2^Department of Health Promotion, Faculty of Physical Therapy, Srinakharinwirot University, Ongkharak Campus, Nakhon Nayok 26120, Thailand

**Keywords:** community healthcare, elderly, online exercise program, online traditional dance, stress

## Abstract

**Objective:** This quasi-experimental research was to assess the effect of online traditional dance exercise program on stress among elderly in Nakhon Nayok province, Thailand.

**Methods:** The elderly living in Nakhon Nayok province were recruited for the study. Multistage random sampling was employed to select participants. There is a total of 27 elderly people classified as experimental Group 15 people (received a 4-week online traditional dance exercise program) and control Group 12 people. Exercise data collected by the SPST20, pre- and postintervention testing was used to measure stress. An independent *t*-test analyses were performed to assess the effectiveness of program interventions.

**Results:** After the program, there were statistically significant differences in mean scores of stress between the experimental and the control group. The level of stress of the elderly in the experimental group had decreased within and between groups after 4-week (*p* value < 0.05).

**Conclusion:** Effectiveness of online traditional dance exercise program on the stress level of the elderly. It can reduce stress levels in the elderly. It could be applied with elderly in the community healthcare setting.

## 1. Introduction

According to data of the United Nations World Population Aging found that Thailand is in the process of transitioning into an aged society completely (Aged Society). The United Nations indicates that any country has a population aged 60 years and over in the proportion of more than 10% of the total population is considered that country is stepping into Aging Society, and will become a “Full Aged Society” (Aged Society) when the proportion of the population aged 60 and over increases to 20%. According to the numbers of Thailand, it is predicted that in 2035, Thailand will enter a completely aging population society with people over 60 years of age exceeding 28% of the total population [1]. This situation is the result of economic development, national development, scientific breakthrough technology, and medicine. These factors caused the population to live longer. Family planning policy or fertility control are causing a rapid reduction in fertility and a steady decline in the mortality level of the population also cause the number and proportion of the elderly population of Thailand to increase rapidly. As a result of a country entering an aging society, the factors of production in terms of labor decrease, the movement of foreign workers has increased, less national income, and expenditure budget increase while the revenue budget is reduced. The government must support the welfare budget for the elderly more. Furthermore, there are more social problems such as the elderly being abandoned, poor mental state, and physical deterioration need to be taken good care of, when entering the elderly, there will be many changes in various aspects such as psychological, physiological, social and economic aspects. Moreover, the stress problem is the most common mental health which is founded among the elderly, and it is caused by physical health problems, and psychological problems such as loss, disappointment, misunderstanding among family members, or social problems various economy. If the elderly has more chronic stress problems, it will negatively affect their physical and mental health, until it may even cause serious physical illnesses such as heart disease, vascular disease, gastrointestinal disease, or various immune-related diseases. The effects of chronic, or long-term, stress can be harmful on their own, but they also can be activated in response to stress in older adult, contributing to increased vulnerability to depression [2–5]. It may also be a psychiatric disease, such as schizophrenia or can be fatal lead to the suicide in order to avoid problems.

Several research studies have found that exercise can help reduce stress because exercising helps to control emotions or stress with the effect of causing happiness and gain resistance to stress, depression, and sadness and reduce sensitivity to stress [6]. It also has the effect of modifying the function of the adrenal glands which secrete hormones that affect the functioning of the midbrain, resulting in behavioral changes to stress. Generally, when stress is occurred, it will make the level norepinephrine decreases but regular exercise will increase the level of norepinephrine. Moreover, noradrenergic effect promotes antistress effect, and exercise also helps the release of endorphin which is opioid substance that help promotes good mood [7–9]. Alleviation by using Thai North-east traditional dance is the other kind of relief; it is an aerobic exercise because it is an activity that moves different parts of the body, including the torso, arms, legs, and hands in sync along with the rhythm of the music [10–13]. It will result in the work of the heart, lungs, blood vessels, and blood circulation system more efficiently because of the body releases endorphins (endorphin) to reduce the stress level. Exercising by using Thai North-east traditional dance not only improve the physical performance, but also affects the quality of life and mentality of the volunteers. Due to Thai North-east traditional dance, it is a kind of aerobic exercise with rhythmic, melody, and choreographed of dances that can entertain and make subjects happy and increase quality of life as well [14]. In the area of Nakhon Nayok Province, there are Thai Puan people living. Which has traditions that are consistent with the culture of Northeastern Thailand, such as the parade of rockets, cat lady Thai traditional dance, dragging boards, Su Khwan Khao (Thai blessing ceremony or Bai Sri tradition to present rice), Kam Kiang tradition, gathering to harvest the rice (Long Khaek Kiao Khao or Hao) tradition, Boon Khao Jee tradition, eat Khao Mao tradition, and Boon Khao Lam tradition [12, 13]. Therefore, it is in line with the existing Thai Puan culture and interesting to put in the exercise program.

At present, due to the situation of the epidemic of COVID-19, there is still violence. Therefore, the government has issued risk reduction measures to prevent infection in order to monitor and prevent the spread of COVID-19, causing the need to have social distancing. Social distancing caused some places to shut down, such as parks and fitness centers and making people unable to live a normal life. Therefore, organizing activities in an online format is something that can replace normal life well, due to the use of online communication reduces the risk of a COVID-19 outbreak.

Common barriers to engaging in exercise include time constraints, lack of access to facilities or equipment, and a limited knowledge of safe, effective physical activity methods. Contemporary interventions leveraging technology employ methods such as web-based platforms, text messaging, smartphone applications, and social media-fused podcasts [15, 16]. Online exercise is gaining traction, offering advantages over onsite exercise in terms of accessibility, scalability, cost-efficiency, and adaptability. This development has not only facilitated but also enhanced the efficacy of health promotion interventions. The growing acceptance of these interventions is largely fueled by increased access to and ownership of tech gadgets, particularly smartphones [17]. Considering that online exercise is easily accessible to populations worldwide, our results widely support the validity of online exercise. Although online exercise is undoubtedly useful and important, chronic adaptations to online exercise and the corresponding effect on mental health and well-being remain unclear.

Online exercise to reduce stress for the elderly is very appropriate. Because the elderly are a high-risk group, they contract various diseases and die more easily than other populations. Including difficulty moving, do not want to leave the house (homebound elderly). Therefore, online exercises for seniors are helping to reduce the incidence of disease. It also helps seniors who have mobility problems; there is no need to travel to the gym to exercise. It helps reduce stress, reduce travel expenses, and make exercising more comfortable as well [18]. The elderly prefer to use LINE application as a social media the most because it is easy to use, can view pictures, short video clips, and links to watch video examples of various exercises. This study used the application to introduce the elderly to exercise online and used Line group video calls to encourage the elderly to exercise in groups online [19–21].

For that reason, the researcher is interested in developing an exercise program in the elderly by using traditional dance to study the effectiveness of a dance exercise program on stress levels in the elderly by doing it online, since no studies have been conducted to study exercise in the elderly with online form.

## 2. Objectives

The aim of this study was to investigate the effect of an online traditional dance program on the stress the elderly.

### 2.1. Conceptual Framework

Independent variable was the exercise program with Thai North-east traditional dance.

**Figure 1:**
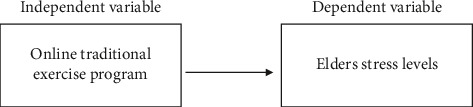
Conceptual framework of the study.

Dependent variable was the stress level of the elderly (see Figure 1).

## 3. Methodology

### 3.1. Population and Sample

This study was quasi-experimental research using the intervention and the control groups. Participants were elderly aged 60 years or older living under responsibility of Ban Khmer Fung Tai Subdistrict Health Promotion Hospital, Nakhon Nayok province. There is a Thai Puan culture. The inclusion criteria were: aged 60 years and over, both male and female, believe in Buddhism, able to use chat line application, living in the area of study, willing to participate throughout the program, no mental health problem, no medical contraindications to exercise brain injuries. Calculate the sample group according to previous studies [22–24]. There were 27 volunteers in this experiment. The researcher divided the sample into 2 groups: an experimental group of 15 people and a control group of 12 people. This process ensured that they were fully aware of the study's potential benefits, and risks. Eligibility screening process helped to reduce the likelihood of physical harm. However, no adverse events were observed. The study started in March 2022 to January 2023. By means of bringing the pretest of stress scores from all samples divided into 2 group which are the experimental group and the control group by alternating left to right ordering method, ascending and descending in order to minimize the difference in scores between the two groups. In our study, we employed strategies to ensure blinding and minimize bias. Such as (1) the participants were unaware of the objective of the experimentation and (2) who administered the treatments were not involved in the collection or analysis of outcome data. This prevented them from potentially influencing the outcome assessments. The key to reducing bias in outcome assessments was blinding the outcome assessors. The outcome assessors were completely blind to the participants' treatment conditions. To achieve this, assessments were conducted using anonymous codes for participants, ensuring that the assessors did not know to which group the participants belonged. Additionally, all personnel involved in the study received a periodic monitoring and audits to ensure that blinding procedures were strictly adhered to throughout the study. These measures helped to minimize the risk of bias and ensure that the outcome assessors remained unaware of the participants' treatment conditions. The participants were unaware of the study hypotheses, but inevitably knew what type of training they were following, which could represent, even indirectly, a limitation to the blinding procedure.

### 3.2. Research Tools

1. This study used line official application to introduce the elderly to exercise online and used Line group video calls to encourage the elderly to exercise in groups online. Exercise program with Thai North-east traditional dance which was based on a literature review and an expert review of the program's suitability and allow volunteers to participate online via LINE Official.

The contents of the menu consisted of 4 menus as follows:

1. Schedule of activities.
2. Video clips of dancing exercises.
3. Infographics of dancing exercises and their benefits.
4. Stress assessment form.

If the volunteer has questions, they can ask for more information through the chat channel at any time. A total of nine poses, divided into exercise for set one of five poses and exercise for set two of four poses for volunteers to be able to switch sets each time, which increase the interest of the program, and reduce the boredom of having to exercise at same posture throughout the period of joining the program for 12 times (total of 4 weeks, 3 times a week, and 40 min each time). Each time, the details of the program are as follows:

1. 5 min warm-up
2. Thai North-east traditional dance which consists of nine exercise postures, divided into 2 sets. First set consist of five postures (swaying dance, hand wiping pose, finger slipping pose, Yung Pan second rhythm dance pose, and four-faced Brahma pose). Second set consist of four postures (Seng girl strolls into the field, Seng Swing, Sod Soi Mala, and shy pose).
3. Cooldown for 5 min.

As mention previously, all of the traditional dance program will be made into video clips which the researcher led the exercise along with the music throughout the program.

### 3.3. Data Collection

In this study, stress level was measured via the Suanprung Stress Test-20 (SPST-20) of Public Health, Thailand [25, 26]. The SPST-20 is a 20-item, with options stress scored on a 5-point scale ranging from 1. The total scores for participants ranged from 1 to 100, with higher scores indicating greater perceived stress.

The SPST-20 was used to assess the stress of the elders, and both in the experimental group and the control group by collecting data twice, before and after participating in the exercise program with dancing.

### 3.4. Data Analysis

Paired *t* test statistics to compare stress before and after participating in the exercise program of the experimental group. Independent *t* test statistics to compare stress between the experimental and control group. With a significance level set at 5% (*p* < 0.05).

### 3.5. Ethical Considerations

This research was reviewed by the Human Research Ethics Committee, Srinakharinwirot University, number SWUEC-060/2565E. All human research procedures followed were in accordance with the ethical standards of the committee responsible for human experimentation (institutional and national) and with the Helsinki Declaration of 1975, as revised in 2013. Participants were also given a thorough orientation before the start of the study, which included detailed instructions on the training protocols, proper use of equipment, and safety measures. The researcher obtained written informed consent from each participant prior to study initiation. Monitoring the participants' health and well-being throughout the intervention was an ongoing process. In case of adverse events, we had a clear protocol for immediate response, including providing first aid and arranging professional support. Participants would have received ongoing support and referrals if needed to ensure full recovery. They were also assured that participation was voluntary and that they could withdraw from the study at any time without any repercussions.

## 4. Results

### 4.1. General Information

According to Table 1, the general information of the sample group consisted of 27 people which were divided into 15 experimental groups. Most of the participants aged between 60 and 69 years, with 14 people, representing 93.33%, followed by the elder who aged 70 years and over, with one person which represent 6.67%. Other participants were 4 males or 26.67%, followed by 11 females which represent 73.33% and *l* person have BMI less than 18.5 which equal to 6.67%, 4 people have 18.5–22.90 BMI which represent 26.67%, and 10 people have more than 23 BMI which amount is 66.67%.

Twelve people from the control group, most of the participants were aged 70 years or over in amount of 7 people represent 58.33%, followed by the elders in the age range of 60–69, 5 people representing 41.67%. Other participants are 7 males which are 58.33% and 5 females in amount of 41.67%, 6 people are having a BMI of 18.5–22.90 which represent 50%, and other 6 people are having a BMI greater than 23 which represent 50%. There is no significant difference between experiment and control group (Table 1).

### 4.2. Stress of the Sample Group

The stress level of the experimental group before participating in Thai North-east traditional dance exercise program, most of them were moderately stressed, with 9 people, representing 60%, followed by high levels of 3 people, representing 20%, low level of 2 people, representing 13.33%, and severe level for 1% which represent 6.67%. After the experimental group participated in the dance exercise program, it was found that most of the experimental groups had moderate stress of 12 people, representing 80%, and 3 people in low levels which represent 20%, respectively.

### 4.3. The Effect of Thai North-East Traditional Dance Exercise Program on Stress Levels

According to Table 2, it was found that the study of data from a sample of 27 people by comparing the stress of the elderly before and after joining the exercise program with Thai North-east traditional dance. From the stress scale of Department of Mental Health (SPST-20) in amount of 20 items, it was found that the stress of the elders in the experimental group before participating in the Thai traditional dance exercise program was 37.53 ± 13.88 and after participating in the program was 25.47 ± 3.25, which were significantly different (*p* value = 0.001) at the 95% confidence level and the stress of the elders in the control group before participating in the dancing exercise program was 32.92 ± 7.53 and after participating in the dancing exercise program was 34.50 ± 13.91, which the difference was not statistically significant (*p* value = 0.596) at 95% confidence level.

Stress of the elderly in the experimental group and the control group before joining the exercise program by dancing, there was no statistically significant difference (*p* value = 0.311), and the stress of the elders in the experimental group and the control group after joining the exercise session with Thai traditional dance, the result was statistically significant difference (*p* value = 0.048) at 95% confidence level.

## 5. Discussion

Exercise, defined as any movement increasing energy expenditure above resting levels, a planned physical activity, is known to improve health and mind across elderly and health conditions [27, 28]. Due to Thai North-east traditional dance, it is a kind of aerobic exercise with rhythmic, melody, and choreographed of dances that can entertain and make subjects happy and increase quality of life as well [29]. This study used line official application to introduce the elderly to exercise online and used Line group video calls to encourage the elderly to exercise in groups online. In addition, the elderly can use this LINE official to talk and exchange information. This shows that online exercise programs, which have become online communities, are very useful and appropriate for the elderly [19–21].

The stress scores of the control group and the experimental group were not significantly different from the pre-exercise. But after excising with online traditional dance program, it was found that the stress scores of the experimental group decreased and were statistically significant different from the control group (*p* value = 0.048). In the view of fact that the participants satisfied and liked the exercise program with Thai North-east traditional dance by commenting that the dance exercise program has enjoyment music and fun rhythm which cause reduction of the stress level in the elders and also give a better metal health. The research found that the promotion of mental health in the elder by moving organs along with the music or dance can help promote mental health in the elder [9, 13]. Thai North-east traditional dance moves accompanying with Luk Thung or Thai country songs cause fun, enjoyment and relieve stress, also reduce the risk of depression and improve mental health in the elders [9]. In addition, stress causes the pituitary gland to be stimulated, this causes the adrenal glands to release more cortisol which can cause a number of physical symptoms that vary from person to person, such as headache, back pain, fatigue. If the person is faced with severe stress, it can result in the death of a person. Due to the failure of the body's system, such as people with diabetes, they already have a congenital disease and also if severe stress occurs, the cortisol hormone will stimulate blood sugar levels to rise or fall in abnormally low and can cause shock. Therefore, the exercise program was designed in order to reduce stress levels in the elders, due to the exercising for 30 min or more is able to reduce the secretion of the hormone cortisol and affecting in decreasing the stress levels. Above-mentioned is consistent with the research on the effects of exercise programs on depression and cortisol hormone among adolescent girls with depression [7–9, 30–32].

The study of the effectiveness of this online traditional dance exercise program was successful because while the participants are participating in the program, the researcher has communicated, followed up and supervised the research participants regularly. In addition, the exercise program online with the Thai North-east traditional dance is also interesting because Thai North-east traditional dance posture in the program is consistent with the culture of the elderly in the area, which made the research participants well cooperate in exercising program [10–13]. Moreover, the highlight of the dance exercise program with Thai North-east traditional dance is the preparation of the program in an online format, and this is in line with the current situation of the COVID-19 epidemic.

Online exercise programs via Line official are programs that the elderly like because it is easy and convenient to use. The elderly can exercise while they are at home. It saves on travel costs. It is suitable for the elderly who are homebound and have difficulty traveling. In addition, the elderly can use it as a channel to talk to many friends in the Line group as if they were meeting each other. This is another reason why online exercise programs can help reduce stress in the elderly [19–21].

According to this study, it can be concluded that the exercise program with online Thai traditional exercise program can be applied to elderly in the community. Because it makes the elders have less stress and depression [2–5] and also promote the health of the elders in many ways, such as physical, mental, social, and intellectual. In addition, reduced stress levels result in a better quality of life for the elderly. And decrease ALY (disability-adjusted life year) and increase HALE (health AL expectancy) too [33].

### 5.1. Limitation of This Study

This study is a quasi-experimental study with a control group and a comparison group. However, the sample size may be small. Therefore, in the next study, the sample size should be increased to confirm the results of the study.

## 6. Conclusion

The quasi-experimental research was to assess the effect of online traditional dance exercise program on stress among elderly. According to the stress scale, it was found that the stress of the elders in the experimental group before participating in the dance exercise program was 37.53 ± 13.88 and after participating in the dance program was 25.47 ± 3.25, which were significantly different (*p* value = 0.001). Moreover, the stress level of the elders in the control group before participating in the Thai North-east traditional dance exercise program was 32.92 ± 7.53 and after participating in the exercise program was 34.50 ± 13.91, which were not statistically significant differences (*p* value = 0.596). In conclusion, it can be concluded that the Thai North-east traditional dance exercise program affects the stress level of the elders, and also be able to reduce stress level in seniors.

### 6.1. Research Recommendations

1. Other aspects of the assessment form should be used to further study the effects of Thai North-east traditional dance exercise program.
2. Improve the program to be culturally diverse by incorporating dance moves and lyrics from other regions.
3. Improve the video media by dividing it into episodes with appropriate duration in order to provide more comfortable to the research participants during the exercise.

## Figures and Tables

**Table 1:** Demographic information of the subjects.

General information	Experiment group (*n* = 15)		Control group (*n* = 12)	
	*N*	%	*N*	%
*Age (year)*				
60–69	14	93.33	5	41.67
≥ 70	1	6.67	7	58.33

*Sex*				
Male	4	26.67	7	58.33
Female	11	73.33	5	41.67

*Body mass index*				
< 18.5	1	6.67	0	0
18.5–22.90	4	26.67	6	50
≥ 23	10	66.67	6	50

**Table 2:** Presents the mean stress scores before and after joining the program of the subjects.

Online traditional exercise program	Before		After	
	Mean	S.D.	Mean	S.D.
Experiment group *n* = 15	37.53	13.88	25.47⁣^∗^	3.25
Control group *n* = 12	32.92	7.53	34.50⁣^∗∗^	13.91

⁣^∗^(*p* value < 0.05, pare *t* test: compare between before and after of experiment group).

⁣^∗∗^(*p* value < 0.05, independent *t* test: compare between the experimental and control group).

## Data Availability

The data used to support the findings of this study are available from the corresponding author upon reasonable request.
